# Dataset of Deglet Nour date palm bunches for smart harvesting

**DOI:** 10.1016/j.dib.2025.112217

**Published:** 2025-10-30

**Authors:** Ahlem Maghzaoui, Emna Aridhi, Sadok Ben Yahia, Sahbi Bahroun, Abdelkader Mami

**Affiliations:** a Faculty of Sciences of Tunis, University of Tunis El Manar, Campus Universitaire, 2092 Tunis, Tunisia; b Department of Mathematics and Computer Science, University of Southern Denmark, Campusvej 55, 5230 Odense M, Denmark; c Higher Institute of Computer Science, University of Tunis El Manar, Rue Abourraihan Al Bayrouni, 2080 Ariana, Tunisia

**Keywords:** Computer vision, Deglet Nour, Keypoint localization, Object detection, Smart harvesting

## Abstract

This article introduces a comprehensive dataset designed to facilitate smart harvesting applications for Deglet Nour date palms, focusing on two primary computer vision tasks: branch detection and optimal cutting point localization. Collected from various oases within the Kebili governorate of southern Tunisia during the peak harvesting season of 2023, the dataset consists of 5530 images annotated for object detection and 387 images precisely annotated with keypoints. Captured using a combination of DSLR and smartphone cameras, the dataset captures real- world agricultural complexities, including varied lighting conditions and occlusions, which were covered and uncorvered date bunches. Annotations, performed manually using Roboflow, are provided in widely used YOLO and COCO formats to ensure compatibility and facilitate widespread adoption. By offering structured, high quality annotated images, this dataset supports the robust development, training, and evaluation of advanced computer vision and robotic harvesting systems for precision agriculture.

Specifications Table

**Table:** 

Subject	Computer Sciences
Specific subject area	Smart harvesting of Deglet Nour date palms using AI
Type of data	Image (JPEG), Annotations (TXT/JSON), YAML, README, Raw, Raw (10,404+ original images, 11 MP4 videos); Cleaned (2304 selected for date branch detection and 161 selected for optimal cutting point detection); Augmented (5530 images for branch detection and 387 for cutting point detection, generated with Roboflow); Annotated (bounding boxes and keypoints in YOLO and COCO formats)
Data collection	Field photography using a Canon EOS 60D DSLR: Canon EOS 60D: f/3.5, 30 s to 1/8000 s, 18–135 mm focal length and smartphone cameras (Oppo A93, Redmi A9, Nokia 4.2) in date palm orchards: Oppo A93: f/1.8, 1/4 s to 1/10,000 s, 26 mm focal length, Redmi A9: f/2.2, 1/3 s to 1/4000 s, 28 mm focal length, Nokia 4.2: f/2.0, 1/5 s to1/3000 s, 27 mm focal length
Data source location	City: Kebili, Province: Kebili, Country: Tunisia. The oases span approximately 33°42′17.9″N, 8°58′15.4″E. The specific oasis locations are as follows: El Menchia Oasis (33.76814, 8.863779); Bou Abdellah Oasis (33.831292, 8.879205 and 33.823739, 8.899460); Tombar Oasis (33.732547, 8.895292); Telmine Oa- sis (33.715068, 8.916325); El Mansoura Oasis (33.720110, 8.938678); Jemna Oasis (33.559438, 8.982455); Bazma Oasis (33.678791, 9.020788); Bechr Oa- sis (33.787118, 8.780429); Oum Essoumaa Oasis (33.780090, 8.813420); Jazirat Louhichi Oasis (33.772234, 8.881535); El Golaa Oasis (33.506268, 8.972794 and 33.505998, 8.968419); El Aouina Nord Oasis (33.428356, 9.000930).
Data accessibility	Repository name: ZENODO Data identification number:DOI: 10.5281/zenodo.15387868 Direct URL to data: https://zenodo.org/records/15387868
Related research article	None

## 1 Value of the Data

• Purpose and research problem: The dataset was created to enable reliable autonomous harvesting of Deglet Nour date palms by tackling two coupled computer-vision challenges that current public datasets do not address together: (i) robust in-orchard detection of fruit-bearing bunches under real field variability (strong/uneven illumination, shadows, occlusions by fronds and protective bags, variable viewpoints and devices), and (ii) precise localization of the safe, optimal cutting point on each bunch to guide end-effector placement and minimize crop/tree damage. Existing resources for date palms primarily target fruit detection or maturity grading and seldom include anatomically grounded keypoint labels needed for cut planning. Our dataset fills this gap by providing high-quality images with bounding boxes (bunch detection) and four keypoints (Peduncle Base, Cutting Point, Rachis Base, Rachis Tip), in standard YOLO and COCO formats, so researchers can train, benchmark, and transfer models for real-time detection and manipulation in precision agriculture.
• *Realistic, diverse imagery: The dataset comprises 5530 annotated branch images and 387 keypoint images of Deglet Nour date palm bunches, captured in southern Tunisian oases using DSLR and smartphone cameras. It includes multi-angle views with natural variability different scales, lighting conditions (bright sunlight vs. shade), and occlusions by fronds or protective bags. Such in situ diversity reflects the challenges of field conditions and helps ensure that vision models trained on these data will be robust to the environmental variations encountered in real date fruit plantations.*
• *Branch-level localization and cutting-point annotations: Uniquely, this data set provides both branch localization labels (boxed boxes) and precise cutting-point key points in each branch containing fruit. These annotations support advanced robotic harvesting tasks, since an autonomous picker must know what to cut (branch position) and where to cut (the optimal cut point). In related work, accurate keypoint detection is vital for autonomous harvesting systems, and real-time branch detection methods have been actively explored in precision agriculture. By supplying both branch-endpoints and cut-point labels, our data enables the development of algorithms that plan and execute safe, efficient harvest actions.*
• *Enables a range of vision tasks: The labeled images can be used for multiple computer vision and agronomic tasks. For example, one can train object detectors to identify date boxes or branches and regressors to predict cut-point coordinates. Previous date-palm datasets have been used for fruit detection, maturity classification, and yield estimation; our proposed dataset complements these by adding branch and keypoint-level labels. In particular, data support tasks from branch segmentation and harvest decision-making to visual yield estimation (by counting labeled branches per tree), broadening the possibilities for automated monitoring and analysis in date cultivation.*
• *Standardized formats and automation impact: The annotations are provided in common YOLO and COCO formats, lowering the barrier to adoption with existing deep-learning frameworks and enabling reproducible model benchmarking. This facilitates rapid prototyping of vision models (e.g., YOLO-based detectors) under realistic conditions. By filling a critical data gap, the dataset directly advances date-palm automation research: as recent studies observe, new vision datasets can help put agricultural research at an advanced level by robotizing pre- and post-harvesting tasks. Robotic-vision developers and agronomists can therefore leverage these images to design, train, and evaluate intelligent harvesting systems and yield-monitoring tools for date palm plantations.*

## 2 Background

The Deglet Nour variety of date palm is one of the most economically significant cultivars in North Africa, particularly in Tunisia, where it represents a major agricultural export. Traditional harvesting methods are labor-intensive, time-consuming, and pose risks to workers due to the height and density of date palm trees. As agriculture faces increasing demands for productivity and safety, there is a growing interest in applying artificial intelligence (AI) and robotic solutions to automate harvesting tasks.

Computer vision technologies (particularly object detection and keypoint localization) are essential for enabling robotic systems to identify, locate, and interact with specific plant parts in complex, unstructured outdoor environments. However, the development of robust AI models requires access to large, diverse, and high-quality datasets that reflect real-world variability in lighting, occlusion, and plant morphology.

The remainder of this manuscript is organized as follows. Section 1 outlines the objective of the dataset and highlights its main contributions to precision agriculture and robotic harvesting research. Section 2 provides a comprehensive description of the dataset, detailing its structure, annotation formats, and distinctive features. Section 3 describes the experimental design, materials, and methods, subdivided into three parts: Data Acquisition, which details the field collection process and camera specifications; Data Cleaning and Augmentation, which explains the refinement and expansion of the dataset; and Annotation, which presents the labeling methodology and quality assurance procedures.

### 2.1 Objective

The objective of this dataset is to accelerate the development of intelligent harvesting systems for Deglet Nour date palms by providing a large, diverse image collection annotated for both object detection (fruit branches) and robotic manipulation (precise cut-point) tasks. This serves as a foundation for training and testing vision models that enable automated, precision harvesting in date palm plantations. This dataset enables academic research in precision agriculture, such as keypoint estimation. Research can go deeper in developing lightweight models to predict optimal cutting points under occlusion, advancing robotic manipulation. We can also combine branch detection with cluster density analysis to estimate yield per tree, supporting agronomic studies. For industrial applications, the dataset directly benefits agricultural automation by training real-time systems to identify and cut bunches accurately, reducing labor costs by 30 % (based on analogous studies). It can be integrated with sorting systems to classify fruit maturity (using branch/keypoint context) for optimized supply chains. Finally, we can create 3D reconstructions from keypoints to simulate harvesting scenarios, aiding workflow planning.

Main Contributions:

• Creation of a high-quality annotated dataset specifically for the Deglet Nour variety of date palms, addressing a significant data gap in agricultural AI research.
• Dataset captures real-world complexities (varied lighting conditions, occlusions, protective coverings) to ensure robustness and practical applicability of trained models.
• Detailed annotation for two crucial agricultural computer vision tasks: branch detection (bounding boxes) and cutting-point localization (keypoints).
• Data provided in widely-used formats (YOLO and COCO) to facilitate immediate adoption and easy integration into existing machine learning workflows.
• Public availability via Zenodo to promote transparency, reproducibility, and accelerate advancements in smart agriculture and precision robotic harvesting systems.

## 3 Data Description

**Fig. 1:**
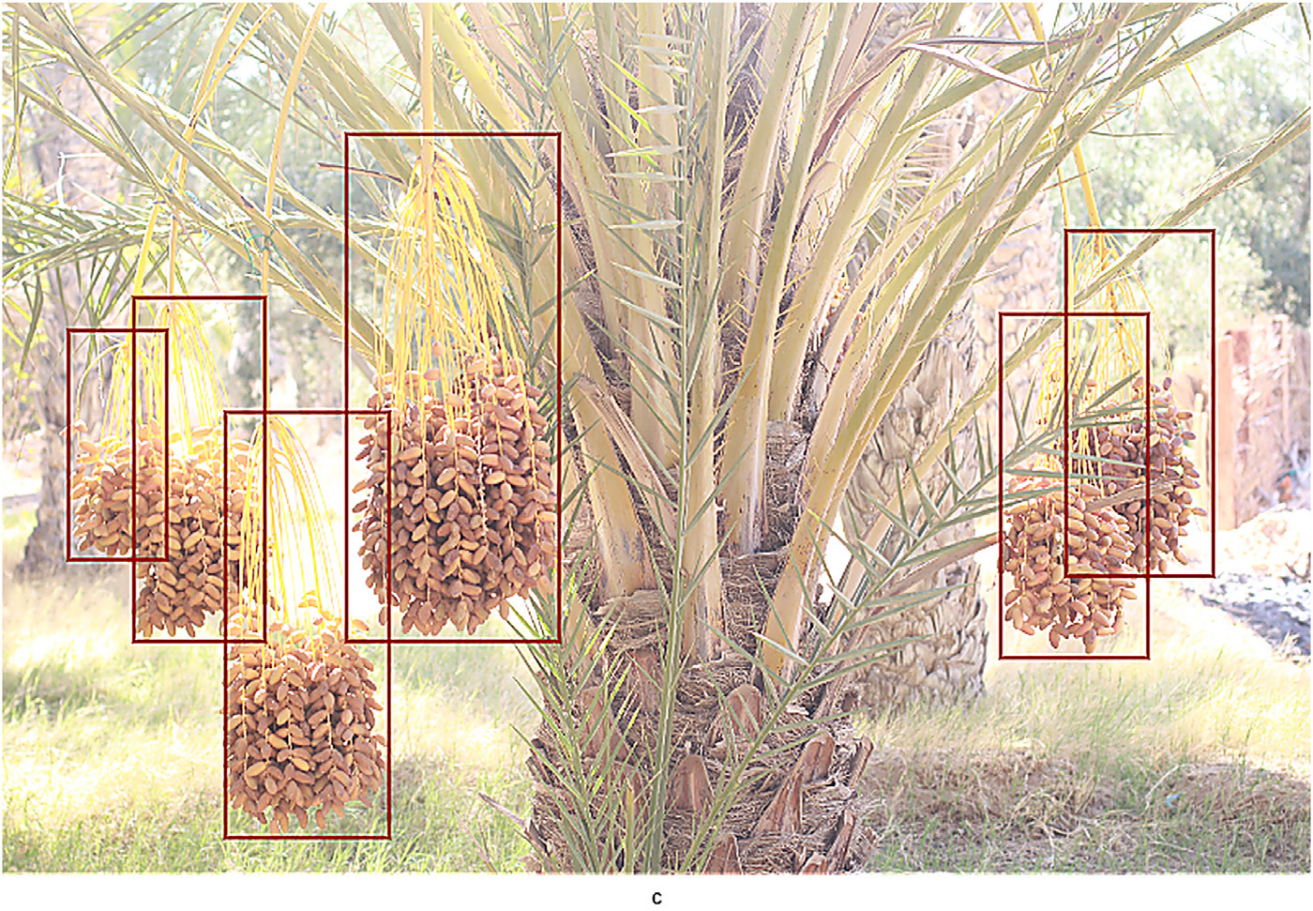
Bounding-box labels for plant branches.

**Fig. 2:**
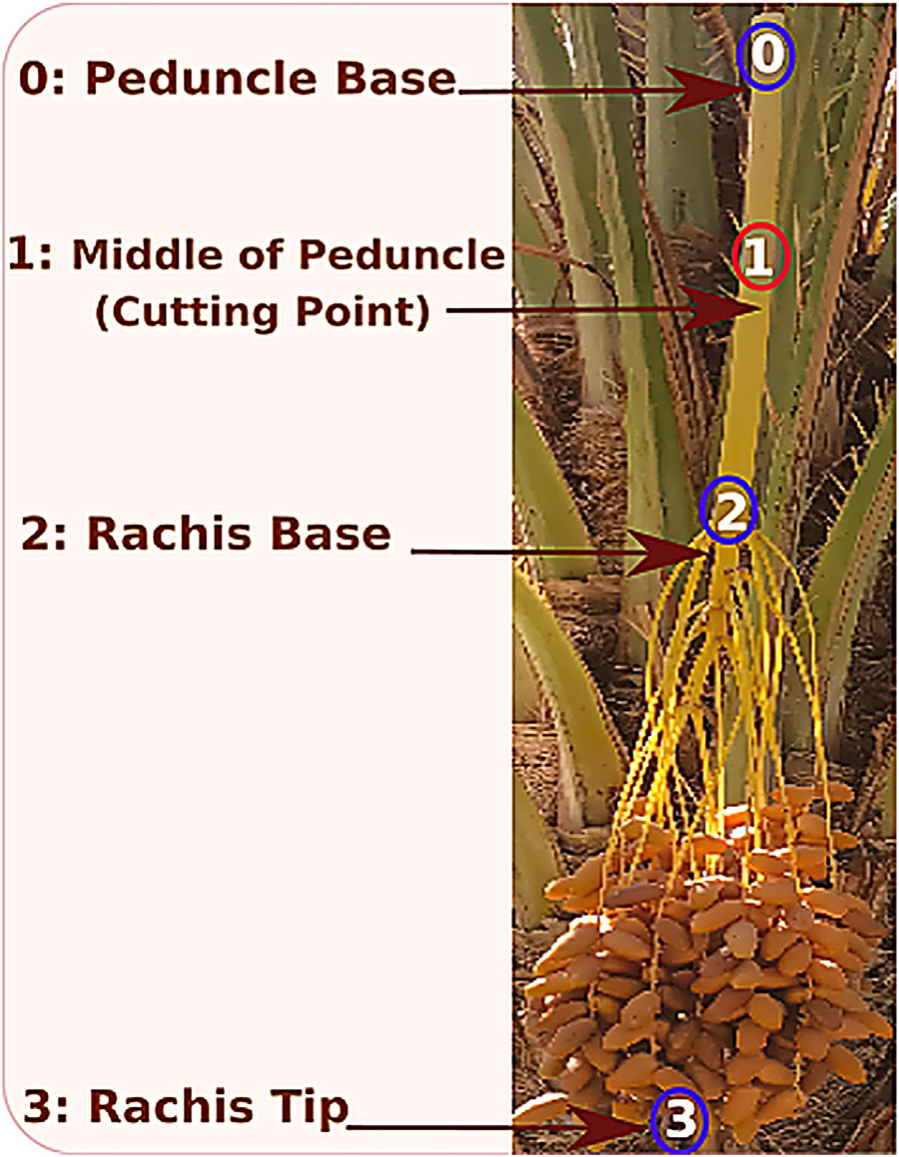
Key point labels for plant branches.

The dataset contains two complementary components tailored for agricultural vision and robotic harvesting research: [1] Branch Detection and [2] Cutting Point Detection. The branch detection component consists of 5530 annotated images with rectangular bounding boxes around each fruit-bearing branch or date bunch, suitable for training object detectors, Fig. 1. The Cutting Point Detection component contains 387 images, each annotated with four keypoints per branch: the Peduncle Base, the Cutting Point (the optimal place to sever the cluster), the Rachis Base, and the Rachis Tip, Fig. 2. These anatomical landmarks indicate where a robotic cutter should place its tool on each fruit cluster

All images were captured in the region of Kebili, in the southwest of Tunisia, using both a Canon DSLR and various smartphones to ensure diversity. The image set spans a range of real-world conditions: different sun angles (bright sunlight to overcast), heavy shade, occlusions by palm fronds or overlapping bunches, and the presence or absence of protective bags on the fruit. This variation simulates the challenges faced by robotic vision systems in the field of smart agriculture. In the branch detection images, each visible target branch (with its fruit cluster) is enclosed by a bounding box. In the cutting-point images, each branch is marked with the four specified keypoints. These tasks align with common goals in precision agriculture computer vision, namely detecting fruit clusters and guiding cut locations for automated harvesters [2]. (For context, other large fruit datasets exist like, the FV40 benchmark with 14,511 images of 40 fruit/vegetable types [3], but none specifically address Deglet Nour palms with cutting-point labels.)

The primary purpose of the dataset is to support machine-learning models for automated harvesting and orchard management. For example, the branch detection subset can train object detectors (such as YOLO or Faster R-CNN) to localize date clusters or branches in orchard scenes, while the cutting point subset can train pose or keypoint-estimation models to predict where a robotic cutter should operate. In practice, a harvesting robot could use a branch detector to find a fruit cluster and then use the cut-off points to accurately place its cutting tool under field conditions. Such integrated capabilities reflect trends in precision agriculture, where vision-based produce localization and cut-point estimation have been actively pursued, Overview of the dataset shown in Table 1.

**Table 1:** Dataset overview.

Task	Classes / Targets	Total images	Recommended split (train / val / test)	Annotation formats	Annotation granularity	File/label types	License
**Branch detection**	1 class: *date-bunch / branch*	**5,530**	**4,839 /461 / 230** (70/20/10)	YOLOv8 (TXT), COCO (JSON)	Bounding boxes	JPG + TXT/JSON (640 640 × 640 (normalized)) **1.2GB**	CC BY 4.0
**Cutting-point localization**	1 class: *branch* with **4 keypoints**	**387**	**339 / 39 / 38** (80/32/16)	YOLOv8 keypoints (TXT), COCO (JSON)	4 keypoints per instance		
**All Raw Dataset**	**Image (.jpg)+ Videos(.MP4)** **48 GB**						

**Fig. 3:**
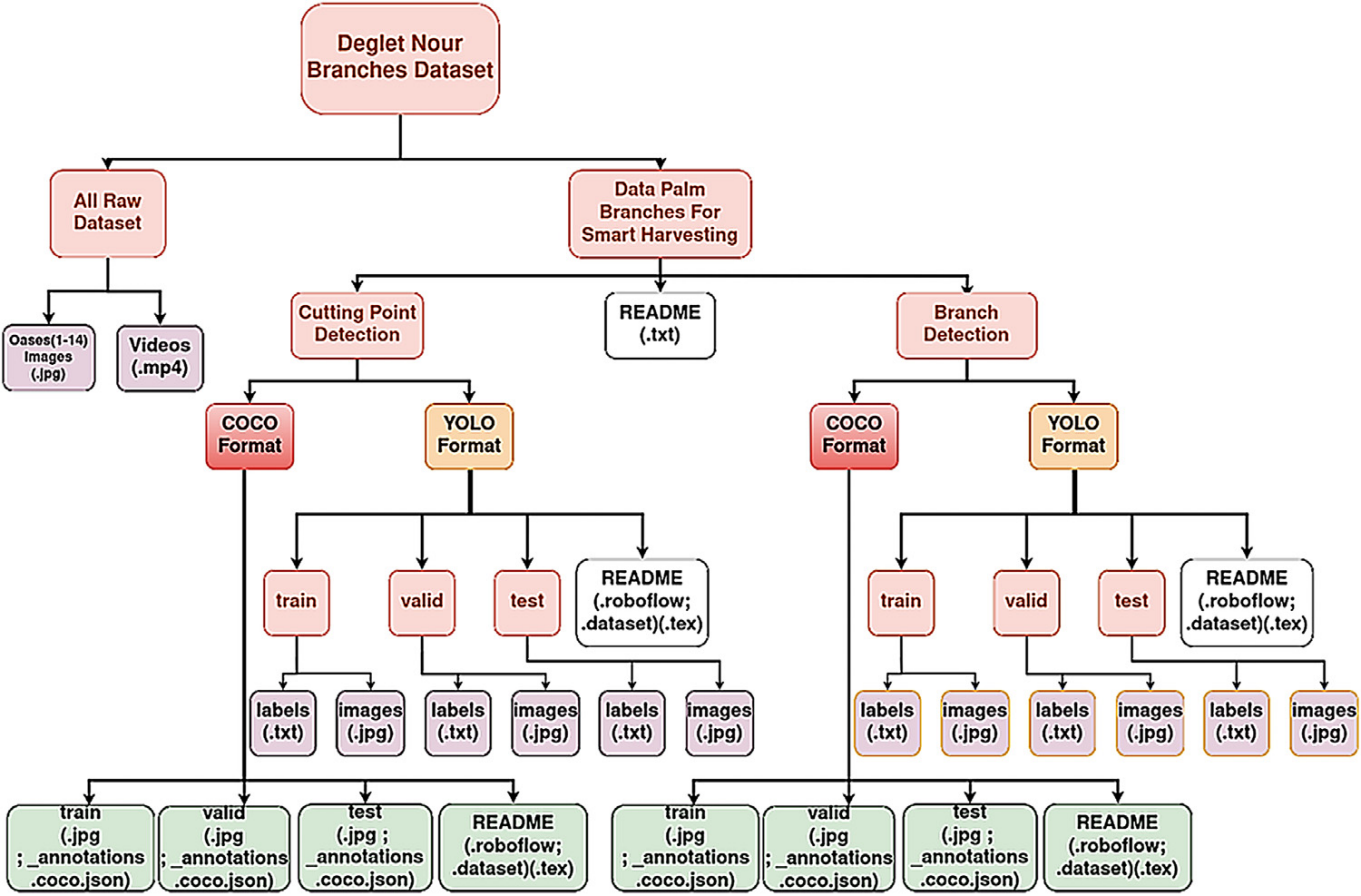
Data collection and folder structure of the dataset.

The Zenodo record is organized into two top-level parts: All_Raw_Dataset (original JPG images per oasis in Oases1–14.zip plus videos.zip) and Data_Palm_Branches_for_Smart_Harvesting (the curated, annotated release). The README documents the folder structure as follows: the curated release contains two task directories branch_detection and cutting_point_detection and each task is provided in YOLOv8 and COCO formats with fixed train/valid/test splits. For YOLOv8, each split includes images/ and labels/ folders with .txt labels using normalized coordinates; for the keypoint task, the four points are ordered PeduncleBase, CuttingPoint, RachisBase, RachisTip. A companion data.yaml lists class names, keypoint schema (where applicable), and relative paths. For COCO, each split contains images/ and annotations.json. Every format folder also provides brief README files (roboflow/dataset) that document export settings, preprocessing/augmentation steps, and any format-specific notes. The overall directory layout of the dataset is illustrated in Fig. 3.

## 4 Experimental Design, Materials and Methods

**Fig. 4:**
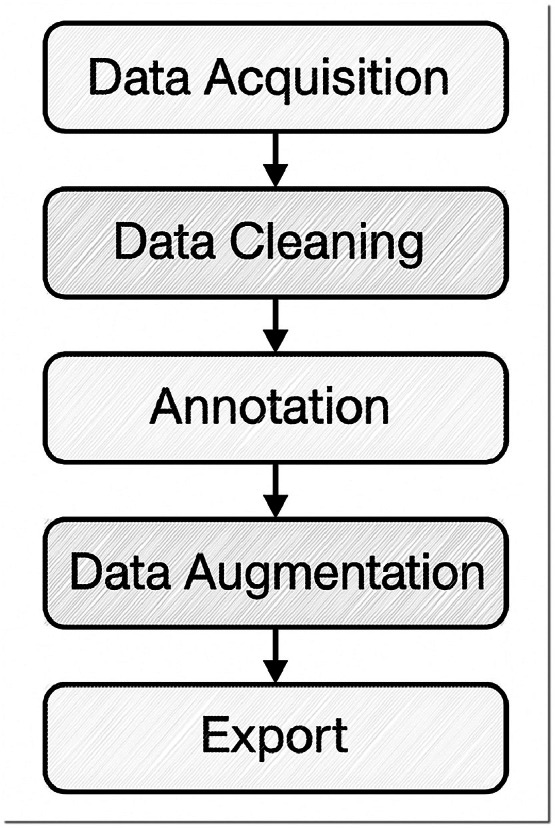
workflow.

Workflow Summary: The dataset creation workflow (Fig. 4) consists of sequential stages: [1] Data Acquisition, where images and videos of Deglet Nour date palm bunches were collected from 14 oases using DSLR and smartphone cameras; [2] Data Cleaning, in which duplicate or blurry images were manually removed through visual inspection by the principal investigator; [3] Annotation, performed in Roboflow (May 2025 stable version), with bounding boxes for branch detection and four keypoints per instance for cutting-point localization; (4) Data Augmentation, applying rotation, scaling, and flipping to increase variability and robustness; and (5) Export, where curated datasets were generated in YOLOv8 and COCO formats with fixed train/validation/test splits, each accompanied by metadata and README documentation for reproducibility.

**Fig. 5:**
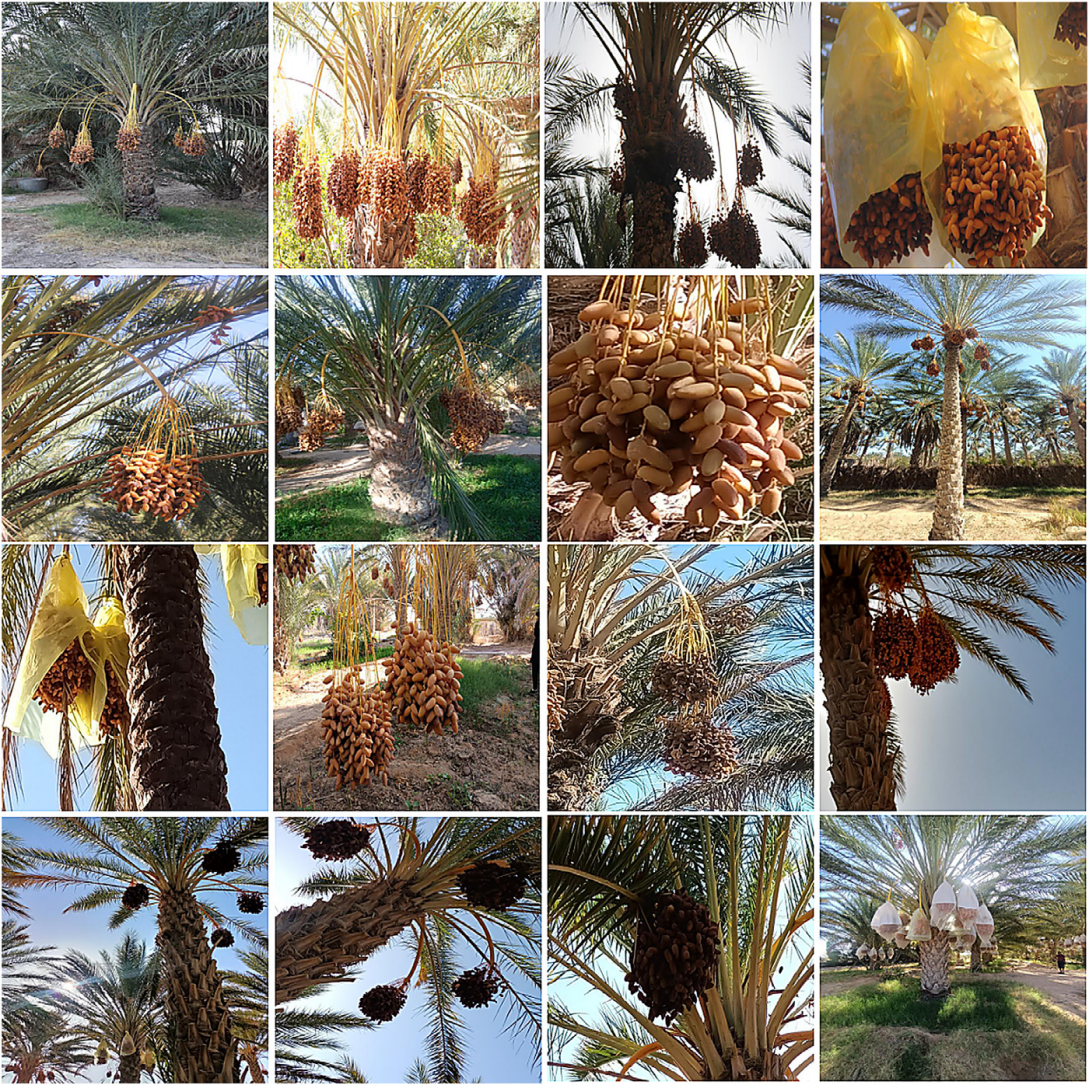
Dataset.

Data acquisition: Images were collected during the peak harvest season of 2023 (from August to September 2023) in the Kebili region. 14 oases were selected (El Menchia, Bou Abdellah (two sites), Tombar, Telmine, El Mansoura, Jemna, Bazma, Bechr, Oum Essoumaa, and Jazirat Louhichi, El Golaa (two sites), El Aouina Nord) to capture geographic and environmental diversity. Field photography was conducted under natural illumination using a Canon EOS 60D DSLR and smartphone cameras (Oppo A93, Redmi A9, Nokia 4.2). These devices provided varying resolutions, focal lengths, and exposure settings and were used to capture the complexity of real agricultural scenes, including bright and diffuse lighting, as well as leaves and fruits occlusions. Both covered (bag-protected) and uncovered date bunches were included, reflecting common plantation practices, Fig. 5.

Data cleaning and augmentation: An initial collection comprised 10,404 raw images and 11 videos (available in the zenodo repository), the dataset was manually curated by the principal investigator. Using direct visual inspection (naked eye), duplicate captures and blurry images were systematically removed. Blurry images were defined as those affected by motion blur, poor focus, or harsh lighting that compromised visibility of date bunch structures. Near-duplicate images of the same bunch from similar angles were also discarded. This manual verification ensured that the final dataset only contained clear, unique, and representative images suitable for annotation and model training. We first removed duplicate, blurry, or irrelevant images, yielding about 8, 000 high-quality frames. We then manually selected final samples: 2, 304 images containing one or more clear fruit-bearing branches for the branch-detection subset and 161 images (usually with one to a few visible branches) for the cutting-point subset. To increase variability and enhance model robustness, we applied three augmentation techniques using Roboflow: rotation, scaling, and flipping. Rotation was introduced to mimic variations in branch orientation and camera angle. Scaling simulated differences in subject distance, ensuring robustness to variable image sizes. Horizontal and vertical flipping reflected the natural orientation variability of date palm branches. These augmentations were selected because they realistically represent conditions encountered in the field while maintaining the biological integrity of the fruit bunch structures. Other augmentations (*e.g.*, color distortions) were not used, as they could compromise the natural appearance of the dataset. Resulting in 5530 images for branch detection and 387 images for cutting-point localization.

Annotation: Annotations were created using the Roboflow annotation platform, specifically its stable release available as of May 2025. This version offers robust annotation tools and supports various formats, ensuring compatibility and reproducibility of the labeling process.

To ensure the accuracy and consistency of the annotations, the principal investigator personally conducted the labeling process. Each image was meticulously reviewed twice to minimize errors and achieve high annotation precision. This rigorous quality assurance approach ensures that the dataset meets the standards required for training and validating AI models in precision agriculture and robotic harvesting applications.

**Fig. 6:**
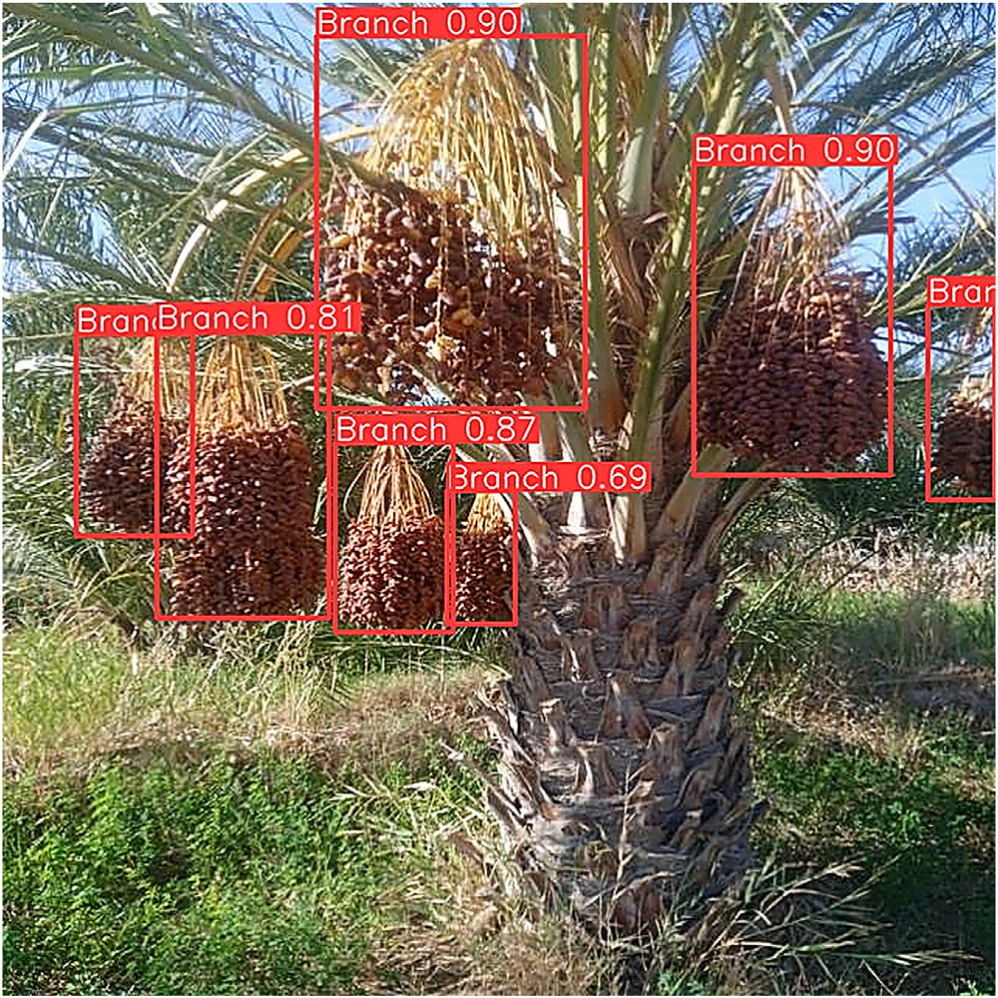
An example of the YOLOv10 detection result.

**Fig. 7:**
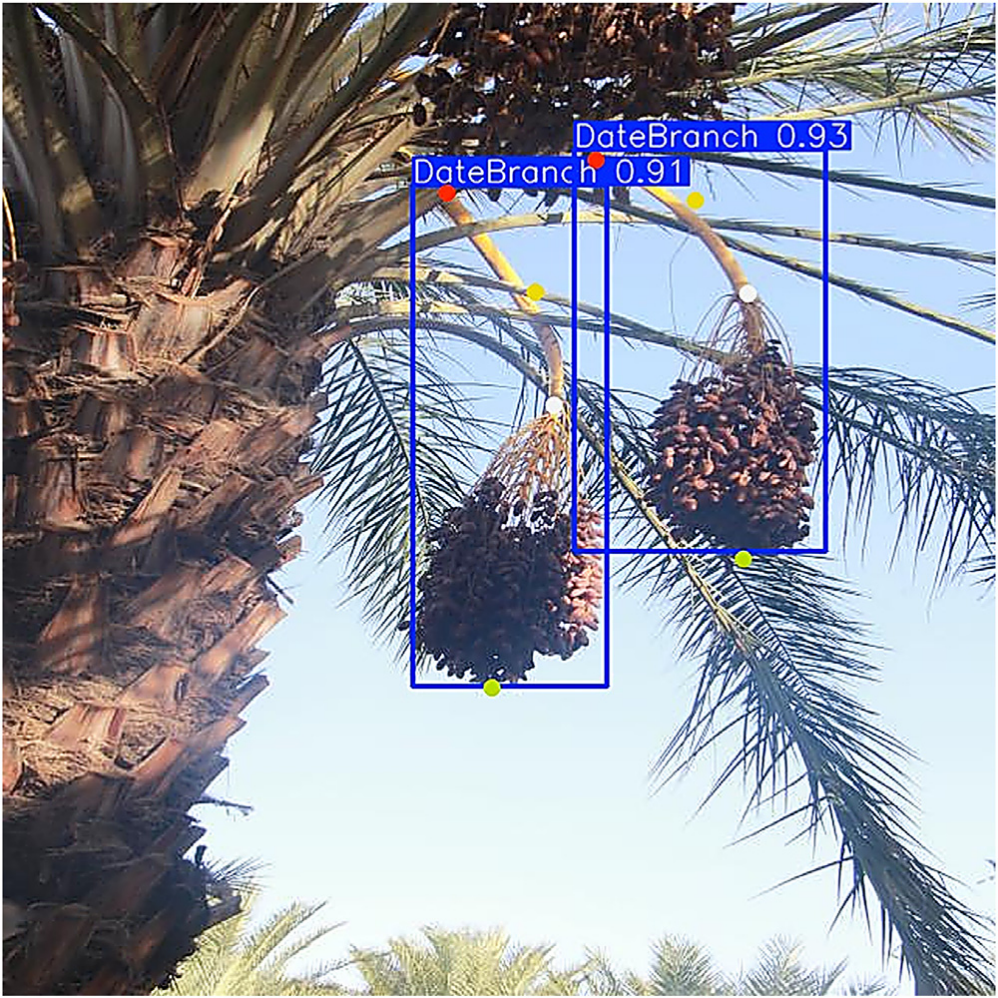
An example of the YOLOv11-Pose detection results.

For branch detection, annotators drew tight rectangular bounding boxes around each Deglet Nour date bunch or fruit-bearing peduncle (Fig. 6). For cutting- point localization, four keypoints were placed on each branch cluster: Peduncle Base, Cutting Point, Rachis Base, and Rachis Tip (Fig. 7), based on established botanical landmarks.

*These annotations were validated and corrected through multiple rounds of checking to ensure consistency.*

This structured and annotated dataset enables the development and benchmarking of vision models and robotic systems to address practical challenges in the harvesting and management of precision date palms. To further support users in leveraging the dataset for machine learning applications, we provide a usage guide and sample code. The dataset can also be accessed programmatically from Roboflow using their API, the manuscript includes a code snippet that demonstrates how to download the YOLOv8 version of the dataset for direct use.

from roboflow import Roboflow

# Initialize Roboflow with your API key

rf = Roboflow(api_key="nePpidh1hVslCdoi2Y6D")

# Access the project and version

project = rf.workspace("ahlem- zxq35").project("datebranchdetection")

version = project.version(1)

# Download the YOLOv8 dataset

dataset = version.download("yolov8")

# For COCO format, use:

# dataset = version.download("coco")

## Limitations

None

## Ethics Statement

This work did not involve the use of human participants, animal experiments, or any data derived from social media. All images in the dataset were collected from agricultural environments with the consent of local farmers. The dataset consists exclusively of visual data depicting date palm trees and does not contain any personally identifiable information or sensitive content

## CRediT Author Statement

**Ahlem Maghzaoui:** Conceptualization, Data curation, Investigation, Methodology, Annotation, Writing–Original. Draft, Visualization. **Emna Aridhi:** Supervision, Methodology, Writing–Review Editing, Validation; **Sadok Ben Yahia:** Supervision, Project administration, Writing–Review Editing, Resources; **Sahbi Bahroun:** Validation, Visualization, Writing–Review Editing; **Abdelkader Mami:** Supervision, Writing–Review Editing.

## Acknowledgements

The authors would like to thank the date palm growers and field workers in the Kebili oases of southern Tunisia for granting access to their farms and supporting the data collection process.

## Data Availability

ZenodoDataset of Deglet Nour Date Palm Bunches for Smart Harvesting (Original data).
